# Amyloid‐Beta Pathology Increases Synaptic Engulfment by Glia in Feline Cognitive Dysfunction Syndrome: A Naturally Occurring Model of Alzheimer's Disease

**DOI:** 10.1111/ejn.70180

**Published:** 2025-08-11

**Authors:** Robert I. McGeachan, Lucy Ewbank, Meg Watt, Lorena Sordo, Alexandra Malbon, Muhammad Khalid F. Salamat, Makis Tzioras, Joao Miguel De Frias, Jane Tulloch, Fiona Houston, Danièlle Gunn‐Moore, Tara L. Spires‐Jones

**Affiliations:** ^1^ Centre for Discovery Brain Sciences The University of Edinburgh Edinburgh UK; ^2^ UK Dementia Research Institute at The University of Edinburgh Edinburgh UK; ^3^ The Hospital for Small Animals, Royal (Dick) School of Veterinary Studies The University of Edinburgh Edinburgh UK; ^4^ The Roslin Institute The University of Edinburgh Edinburgh UK; ^5^ Department of Pathology and Laboratory Medicine University of California Irvine; ^6^ Scottish Brain Sciences, Scottish Gas Murrayfield Stadium Edinburgh UK

## Abstract

Feline cognitive dysfunction syndrome (CDS; a.k.a. feline dementia) is an age‐related neurodegenerative disorder, comparable to dementia in people, characterised by behavioural changes such as increased vocalisation, altered social interactions, sleep–wake cycle, disorientation and house‐soiling. Although the underlying mechanisms remain poorly understood, pathologies similar to those observed in Alzheimer's disease (AD) have been identified in the brains of aged or CDS‐affected cats, including brain atrophy, neuronal loss, amyloid‐beta plaques, tau pathology and cerebral amyloid angiopathy. Neuroinflammation and synapse loss, other important hallmarks of AD, may also play important roles in feline ageing and CDS, but these are yet to be explored. Several mechanisms of synapse loss have been described in human AD and mouse models of amyloidopathy, including synaptic accumulation of amyloid‐beta and the aberrant induction of synaptic engulfment by microglia and astrocytes. In this study, immunohistochemistry and confocal microscopy were used to examine the parietal cortex of young (*n* = 7), aged (*n* = 10) and CDS‐affected (*n* = 8) cats. Linear mixed effect modelling revealed that amyloid‐beta accumulates within synapses in the aged and CDS‐affected brain. Additionally, in the aged and CDS groups, there was microgliosis, astrogliosis and increased synaptic engulfment by microglia and astrocytes in regions with Aβ plaques. Further, microglia and astrocytes show increased internalisation of amyloid‐beta‐containing synapses near plaques. These findings suggest that amyloid‐beta exerts a pathogenic effect in the feline brain, with mechanisms mirroring those seen in human AD. Importantly, these results support the use of feline CDS as a naturally occurring, translational model of Alzheimer's disease, offering valuable insights into AD pathogenesis and potential therapeutic targets.

## Graphical abstract

McGeachan et al. demonstrate microgliosis, astrogliosis and increased synaptic engulfment by microglia and astrocytes in regions with Aβ plaques in aged cat brains and cats with cognitive dysfunction syndrome (CDS). Microglia and astrocytes also show enhanced internalisation of amyloid‐beta‐containing synapses near plaques. These findings suggest that amyloid‐beta drives pathogenic processes in the feline brain, mirroring mechanisms seen in human Alzheimer's disease.

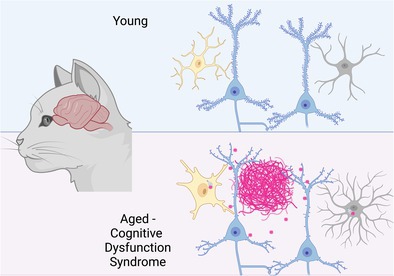

**Abbreviations**
Amyloid‐beta: Aβ/amyloid‐beta
AD: Alzheimer's disease
AIC: Akaike information criterion
ANOVA: Analysis of variance
APP/PS1: Amyloid precursor protein/presenilin 1
ARUK: Alzheimer's Research UK
BIC: Bayesian information criterion
CDS: Cognitive dysfunction syndrome
GFAP: Glial fibrillary acidic protein
IBA1: Ionised calcium binding adaptor molecule 1
LMEM: Linear mixed effects model
mGluR5: Metabotropic glutamate receptor 5
PBS: Phosphate‐buffered saline
PMI: Post mortem interval
PrPC: Cellular prion protein
QQ‐plot: Quantile–quantile plot
RDSVS: Royal (Dick) School of Veterinary Studies
ROI: Region of interest
UK DRI: UK Dementia Research Institute
VERC: Veterinary Ethical Review Committee

## 1 Introduction

Feline cognitive dysfunction syndrome (CDS; a.k.a. feline dementia) is a clinical disorder observed in elderly cats that is similar to human dementia. CDS is characterised by behavioural changes, including increased vocalisation (especially at night), altered social interactions (particularly increased desire for comfort and attention from the owner), changes in the sleep–wake cycle, house‐soiling, disorientation, alterations in activity, anxiety and learning or memory deficits (VISHDAAL). In practice, feline CDS is likely underreported and often unrecognised or underdiagnosed by both owners and primary care veterinary surgeons. There are limited studies investigating the prevalence of feline CDS, but one survey found that 28% of cats aged 11–14 years exhibited at least one clinical sign of CDS, with this increasing to 50% of cats aged over 15 years (Moffat and Landsberg 2003).

The pathophysiology of feline CDS is poorly understood. However, pathologies comparable to those observed in human Alzheimer's disease (AD), the most common cause of human dementia, have been identified in the aged or CDS‐affected feline brain, including brain atrophy and neuronal loss (Levine et al. 1986; Levine 1988; Zhang et al. 2005, 2006; Chambers et al. 2015), amyloid‐beta plaques (Cummings et al. 1996; Nakamura et al. 1996; Brellou et al. 2005; Head et al. 2005; Gunn‐Moore et al. 2006, 2007; Sordo et al. 2021), tau pathology (Head et al. 2005; Gunn‐Moore et al. 2006, 2007; Chambers et al. 2015), and vascular disease, including cerebral amyloid angiopathy (Cummings et al. 1996; Nakamura et al. 1996; Head et al. 2005; Gunn‐Moore et al. 2007). The amyloid‐beta deposits in cats are often less mature and more diffuse than the dense‐core plaques observed in human AD. Instead, these diffuse plaques more closely resemble the amyloid‐beta pathology found in the brains of healthy, aged humans (Gunn‐Moore et al. 2007; Zaletel et al. 2021). Additionally, similar to findings in human AD (Morris et al. 2014), the degree of amyloid pathology correlates poorly with behavioural and cognitive changes in patients with CDS (Head et al. 2005). Furthermore, although studies have shown an age‐related increase in amyloid‐beta accumulation in the feline brain, one study found no significant increase in amyloid‐beta pathology in cats with CDS compared to age‐matched controls (Sordo et al. 2021). This raises an important question: Is amyloid‐beta pathology simply an age‐related incidental finding, or does it actively contribute to neurodegeneration and the signs observed in feline CDS? Determining whether amyloid‐beta exerts a pathogenic effect in the feline brain is becoming increasingly relevant, particularly as monoclonal antibodies targeting amyloid‐beta, which have shown modest success in slowing cognitive decline in people, are being approved for the treatment of AD across the world (Sims et al. 2023; van Dyck et al. 2023).

In human AD, there are pronounced inflammatory changes in microglia and astrocytes, including changes in gene expression, morphology and increased density of these cells, with the most severe changes in the direct vicinity of amyloid plaques (Keren‐Shaul et al. 2017; Henstridge, Hyman, and Spires‐Jones 2019; Habib et al. 2020). While gliosis was initially thought to be largely downstream of pathological changes in neurons, substantial genetic data indicate changes in genes expressed in astrocytes and microglia are associated with an increased risk of AD. Further, recent data link both microglia and astrocytes to synapse loss (Hong et al. 2016; Tzioras, Daniels, et al. 2023; Tzioras, McGeachan, et al. 2023), which is the strongest pathological correlate of cognitive decline (DeKosky and Scheff 1990; Terry et al. 1991; Scheff and Price 2006; Scheff et al. 2007). Understanding and targeting the mechanisms driving glial changes and their contributions to synapse loss are therefore considered a promising therapeutic approach for slowing or halting the progression of AD. Several mechanisms of synapse loss have been described (Tzioras, McGeachan, et al. 2023).

One proposed mechanism is that amyloid‐beta is directly synaptotoxic. Post‐mortem analyses of AD brains (Koffie et al. 2012; Jackson et al. 2019), along with studies using preclinical mouse models with amyloid plaque deposition (Koffie et al. 2009; Pickett et al. 2019), suggest that oligomeric amyloid‐beta accumulates directly within synapses. This accumulation is particularly prominent near extracellular amyloid plaques, where significant excitatory synapse loss is also observed (Koffie et al. 2009, 2012). A recent study using human brain slice cultures supports that amyloid‐beta derived from AD brain tissue is directly toxic to living human synapses (McGeachan et al. 2025). Another potential mechanism leading from pathological amyloid‐beta accumulation to synapse loss is via aberrant induction of synaptic pruning by glia. Synaptic pruning mediated by astrocytes and microglia is an important aspect of normal circuit development (Schafer et al. 2012; Chung et al. 2013; Cserép et al. 2020; Lee et al. 2021). Recently, astrocytes and microglia were observed to engulf synapses in post‐mortem AD brains, with the greatest levels of engulfment in the vicinity of amyloid plaques (Tzioras, Daniels, et al. 2023). Increased synaptic engulfment by glia is not specific to AD and has been described in schizophrenia and other neurodegenerative diseases (Henstridge, Tzioras, and Paolicelli 2019; Tzioras et al. 2021; Dando et al. 2024; McGeachan et al. 2025). Although it is now well established that amyloid‐beta pathology accumulates in the aged and CDS‐affected feline brain (Sordo et al. 2021), the relationship to synapse degeneration has not been explored.

This study investigates whether amyloid‐beta pathology in the feline brain accumulates within synapses and is associated with increased synaptic engulfment by glia.

## 2 Methods

### 2.1 Post‐Mortem Samples and Ethical Approval

The cat brains used in this study were obtained from The Royal (Dick) School of Veterinary Studies. Cats were diagnosed as having CDS if they exhibited behavioural changes consistent with CDS (VISHDAAL criteria) for at least 3 months that could not be attributed to any other medical condition (Gunn‐Moore et al. 2007). CDS‐affected cats (mean age = 16.9 years) were compared to ‘aged’ (mean age = 17.2 years) and ‘young’ (mean age = 4.8 years) control groups with no history of neurologic disease. The CDS and ‘aged’ group were age‐matched (Wilcoxon rank sum test, W = 36.5, *p* = 0.788). Two‐thirds of the cats were female (64%; *n* = 16), one‐third male (32%, *n* = 8), and one cat was of unknown sex. The CDS, age‐matched and young groups were sex‐matched (Fisher's exact test, *p* = 0.34). The demographic details of all cases used in this study, stratified by group, are detailed in Table 1. Consent was obtained from all owners and rescue shelters for the donation of the feline's bodies for research purposes. Ethical approval was gained via the Veterinary Ethical Review Committee from the RDSVS, The University of Edinburgh (VERC 50.17 and 30.20). The brains were fixed in 10% buffered formalin, dissected into anatomical brain regions, and then embedded into paraffin blocks before being cut to a thickness of 4 μm and mounted onto glass slides. Precise post‐mortem interval (PMI) data were not consistently documented across individual cases and therefore could not be reported in detail; however, all tissue was collected and fixed within 4 h post‐mortem, ensuring a short and consistent PMI across the cohort. The focus was to examine the parietal cortex as a previous study has demonstrated the presence of amyloid‐beta within this brain region of aged and CDS feline brains (Sordo et al. 2021). While the parietal cortex was selected to align with previous feline CDS studies, no systematic investigations have been conducted to determine whether amyloid‐beta pathology in cats follows a regionally progressive pattern comparable to Thal staging in AD.

**TABLE 1:** Case details.

Case	Group	Age	Sex	Extracellular Aβ	Pre‐tangles (tau)
DC1001	Young	6	M	−	−
DC1013	Young	4	?	+	−
DC1029	Young	4	F	−	−
DC1033	Young	4	F	−	−
DC1037	Young	6	F	+	−
DC1060	Young	2	F	−	−
DC1062	Young	7	F	+	−
DC1011	Aged	20	M	−	−
DC1012	Aged	25	M	+	+
DC1014	Aged	19	F	+	−
DC1022	Aged	15	F	−	−
DC1023	Aged	16	M	+	−
DC1036	Aged	17	F	+	++
DC1041	Aged	14	M	+	−
DC1043	Aged	15	F	+	−
DC1050	Aged	14	M	−	−
DC1063	Aged	16	F	+	++
DC1005	CDS	18	F	−	++
DC1018	CDS	14	F	−	−
DC1024	CDS	19	M	−	−
DC1030	CDS	16	F	+	++
DC1042	CDS	19	F	+	−
DC1044	CDS	10	F	+	−
DC1049	CDS	19	F	−	−
DC1052	CDS	19	M	−	+++

*Note:* ? = sex unknown. + = mild, ++ = moderate and +++ = severe. The semi‐quantitative tau and amyloid‐beta burdens are from previously published studies (Sordo et al. 2021).

Abbreviations: CDS, cognitive dysfunction syndrome; M, male; F, female.

### 2.2 Immunohistochemistry

The slide‐mounted, paraffin‐embedded tissue was dewaxed in xylene for 6 min and rehydrated in ethanol‐to‐water solutions with decreasing concentrations (100%, 90%, 70%, 50%) for 3 min each to rehydrate the tissue, then thoroughly rinsed with 100% water. Antigen retrieval was performed by immersing slides in 90% formic acid for 5 min at room temperature followed by citrate buffer (Vector labs, H3300) for 3 min in a pressure cooker set to the steam setting. Slides were rinsed in water for 5 min. To reduce autofluorescence (such as that originating from lipofuscin), the autofluorescence eliminator reagent (Merck, 2160) was used as per the manufacturer's instructions. The tissues were outlined using a wax pen, followed by washing with PBS for 5 min, then washed with PBS and 0.3% Triton‐X for an additional 5 min. To eliminate autofluorescence originating from red blood cells, the Vector TrueView Autofluorescence Quenching Kit (SP‐8400‐15) was used as per the manufacturer's instructions. After a 5‐min wash in PBS, the sections were incubated in blocking solutions consisting of PBS, 0.3% Triton X‐100, and 10% normal donkey serum for 1 h. Subsequently, the sections were incubated overnight at 4 °C with the primary antibodies (Table 2). The following day, the slides underwent 2x 5‐min washes using PBS with 0.3% Triton X‐100, a 5‐min wash with PBS, and were then incubated with the secondary antibodies (Table 2) for 1 h at room temperature. To remove unbound secondary antibodies, the slides were washed twice with PBS and 0.3% Triton X‐100, followed by a 5‐min wash with PBS. Finally, one drop of Immumount (Epredia, 9990402) was used in the mounting of the slides to glass coverslips. For each stain, a ‘no primary’ antibody negative control was included.

**TABLE 2:** Antibody information.

	GFAP	4G8	SYN1	IBA1
**Primary antibodies**				
**Species**	Chicken	Mouse (IgG2b)	Rabbit	Goat
**Mono/polyclonal**	Polyclonal	Monoclonal	Polyclonal	Polyclonal
**Company**	Abcam	Biolegend	Merck	Abcam
**Catalogue/lot Number**	cat: #ab4674, lot: GR345545	cat: #800703, lot: B273175	cat: #ab1543P, lot: 3993513	cat: #AB5076, lot: 1053204–1
**Dilution**	1:1000	1:1000	1:500	1:500
**Secondary antibodies**				
**Species**	Donkey anti‐chicken 405	Donkey anti‐mouse IgG 488	Donkey anti‐rabbit 594	Donkey anti‐goat 647
**Catalogue number**	Cat: #703‐475‐155, lot: 155983	Cat: #ab150109, lot: GR3228235	Cat: #a21207, lot: 2747441	Cat: #ab150135, lot: GR3286128‐1
**Dilution**	1:500	1:500	1:500	1:500

### 2.3 Confocal Microscopy and Quantitative Image Analysis

Images were obtained using a 63× oil immersion objective on a confocal microscope (Leica SP8). The imaging parameters were kept consistent throughout each experiment. The tissues were first scanned for amyloid‐beta plaques, and where present, an image stack was acquired at the site of pathology and directly adjacent in a region that did not contain amyloid‐beta pathology but was still within the same cortical layer and only 1 field of view across from the pathology. Each field of view measures 184.7 μm by 184.7 μm. In cases without amyloid‐beta pathology, 10 image stacks were randomly obtained across the six cortical layers of the grey matter. An image was taken of the no primary negative control for each staining batch to ensure there was not non‐specific staining. Using custom ImageJ and MATLAB scripts, the image stacks were segmented, and the percentage volume of the image stack occupied by each channel and the colocalisation between channels was calculated. All custom software scripts used in the image processing and analysis are available on GitHub (https://github.com/Spires‐Jones‐Lab). The experimenter was blinded to the case information during immunostaining, image acquisition and image processing.

### 2.4 Statistical Analysis

Statistical analysis was performed in R studio with R version 4.3.2. The majority of statistical analyses used linear mixed effects models (LMEM)(‘lme4’ R package), as this allowed the testing of whether the group (young vs aged vs CDS) or the presence of amyloid‐beta pathology (at plaque vs no plaque) impacted the variable of interest while controlling for potentially confounding variables, such as age, and including random effects to prevent pseudoreplication. The decision to ultimately include potentially confounding fixed and random effects in the model, and prevent overfitting of the model, was done by inspecting Akaike information criterion (AIC) and Bayesian information criterion (BIC) to assess which model best fits the data. Linear mixed effect models assume linearity, normal distribution of residuals and homogeneity of variance. Linearity was assessed by plotting model residuals against predictors, normality of residuals was checked with a QQ‐plot, and homogeneity of variance was checked by plotting residuals against fitted values. If the model did not meet the assumptions, data was transformed using the method that transformed each individual model to fit the assumptions. Data transformations tested included square root, log, arcsine square root, and Tukey transformation. Post hoc testing was conducted for pairwise comparisons (‘emmeans’ package), with *p* values adjusted using Tukey correction for multiple comparisons. Degrees of freedom were calculated using Kenwood–Roger approximation. Spearman's correlations were run on average values for each case to examine relationships between amyloid beta burdens and synaptic ingestion by glia. Statistics are reported in the results text for main figures and in figure legends for supplementary data.

## 3 Results

### 3.1 Amyloid‐Beta Colocalises With Synapses in the Aged and CDS Feline Brain

**FIGURE 1:**
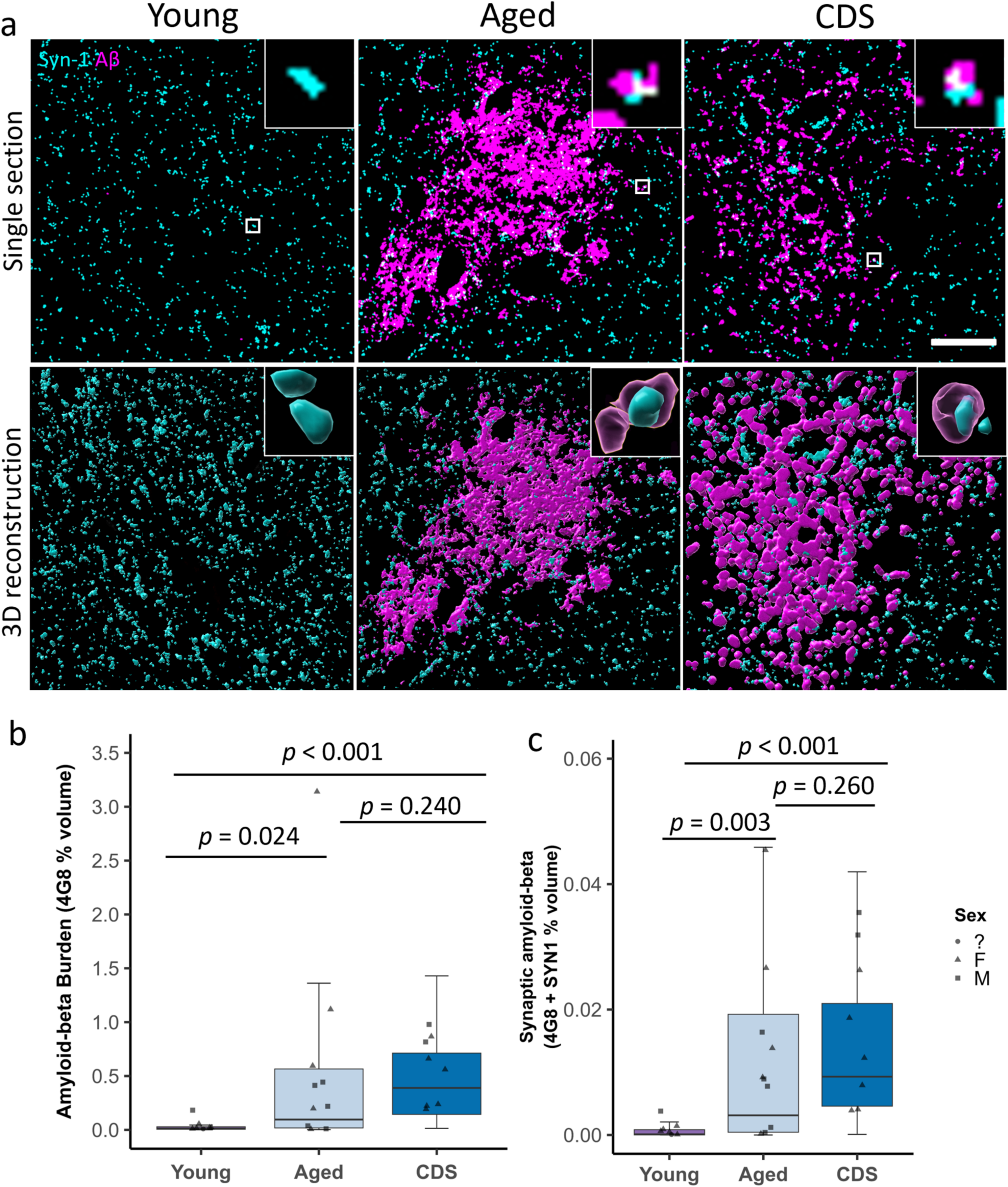
Amyloid‐beta colocalises with synapses in the aged and cognitive dysfunction syndrome (CDS)‐affected feline parietal cortex. (a) Segmented confocal images of young, aged and CDS‐affected feline parietal cortex immunostained for synapses (synapsin‐1, cyan) and amyloid‐beta (4G8, magenta). Scale bar = 20 μm. Inserts = 2 μm × 2 μm. The bottom row of images shows 3D reconstructions generated using IMARIS and have been rotated along the *z*‐axis. Inserts are close‐up images of the colocalisation highlighted by the white box. (b,c) Quantitative analysis reveals an increase in amyloid‐beta burden (b) and colocalisation between amyloid‐beta and synapses (c) in the aged and CDS groups when compared to young controls. Boxplots show quartiles and medians calculated from each image stack. Data points represent case means (sex unknown = circles, females = triangles, males = squares). Statistics: LMEM (variable ~ Group + 1|case). *p* Values were calculated from post hoc testing with Tukey correction for multiple comparisons.

**FIGURE 2:**
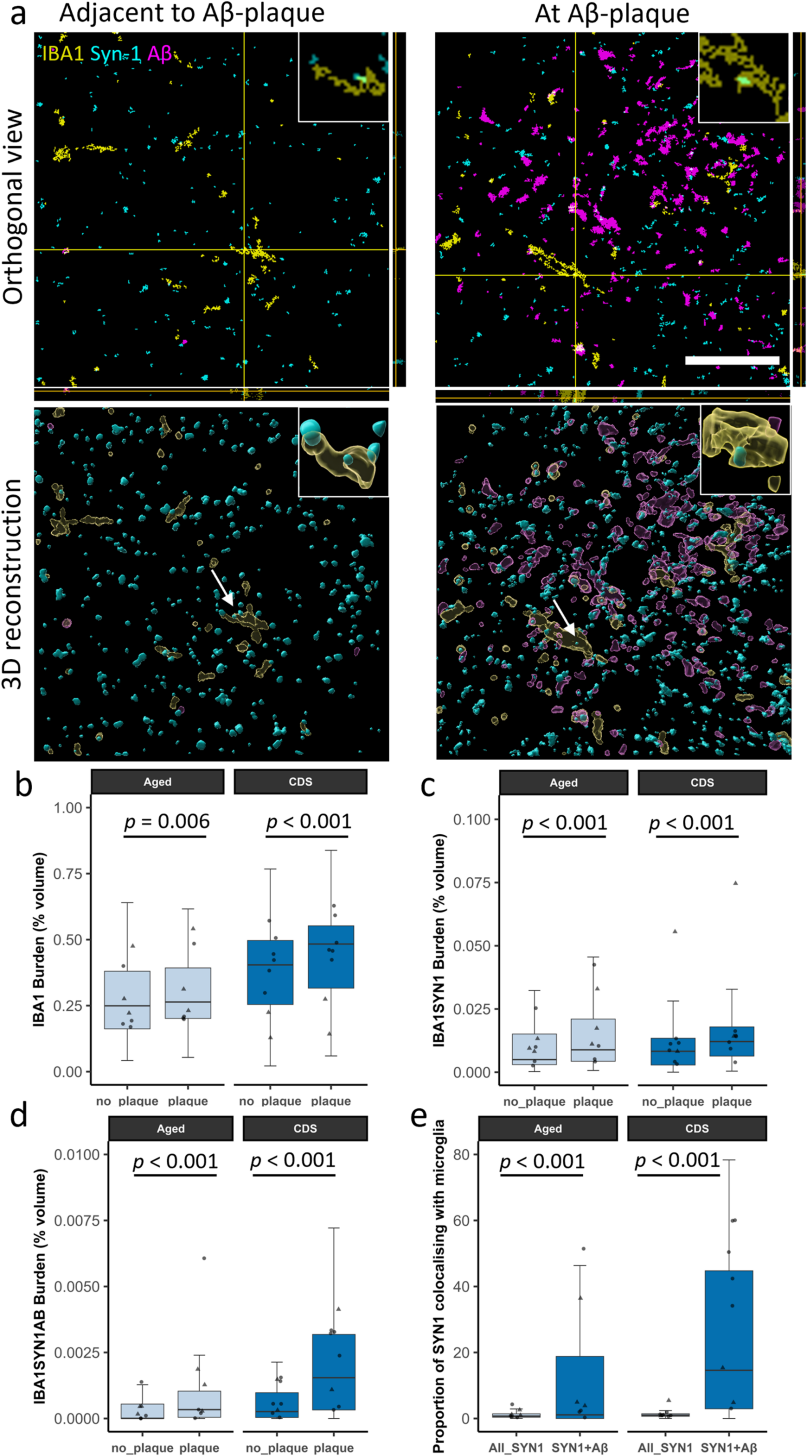
Amyloid‐beta pathology induces increased synaptic engulfment by microglia in the feline brain. (a) Segmented confocal images of cognitive dysfunction syndrome (CDS)‐affected feline parietal cortex, showing a region with amyloid‐beta pathology and a paired image acquired approximately one field of view away in the same cortical layer in an area without pathology. Tissue was immunostained for synapses (synapsin‐1, cyan), amyloid‐beta (Aβ, magenta) and microglia (IBA1, yellow). Scale bar = 20 μm. Insets = 5 μm × 5 μm. The top row of images shows orthogonal views. The bottom row of images shows 3D reconstructions generated using IMARIS and have been rotated along the *z*‐axis, to further demonstrate that synaptic protein is within microglia. Inserts show close up of colocalisation highlighted by arrow. (b–e) Colocalisation analysis reveals that the image stacks acquired in regions with amyloid‐beta pathology show increased microgliosis (IBA1 burden) (b), colocalisation between microglia and synapses (IBA1 and Synapsin‐1 burden) (c), and triple colocalisation between microglia, synapses and amyloid‐beta (IBA1, synapsin‐1 and amyloid‐beta burden) (d). Finally, when analysing all image stacks, synapses colocalising with amyloid‐beta are more likely to be engulfed by microglia (e). Boxplots show quartiles and medians calculated from each image stack. Data points represent case means (females = circles, males = triangles). Stats = LMEM (Variable ~ Plaque_vs_no‐plaque*Group + 1|Case/ROI_number). *p* Values were calculated from post hoc testing with Tukey correction for multiple comparisons.

**FIGURE 3:**
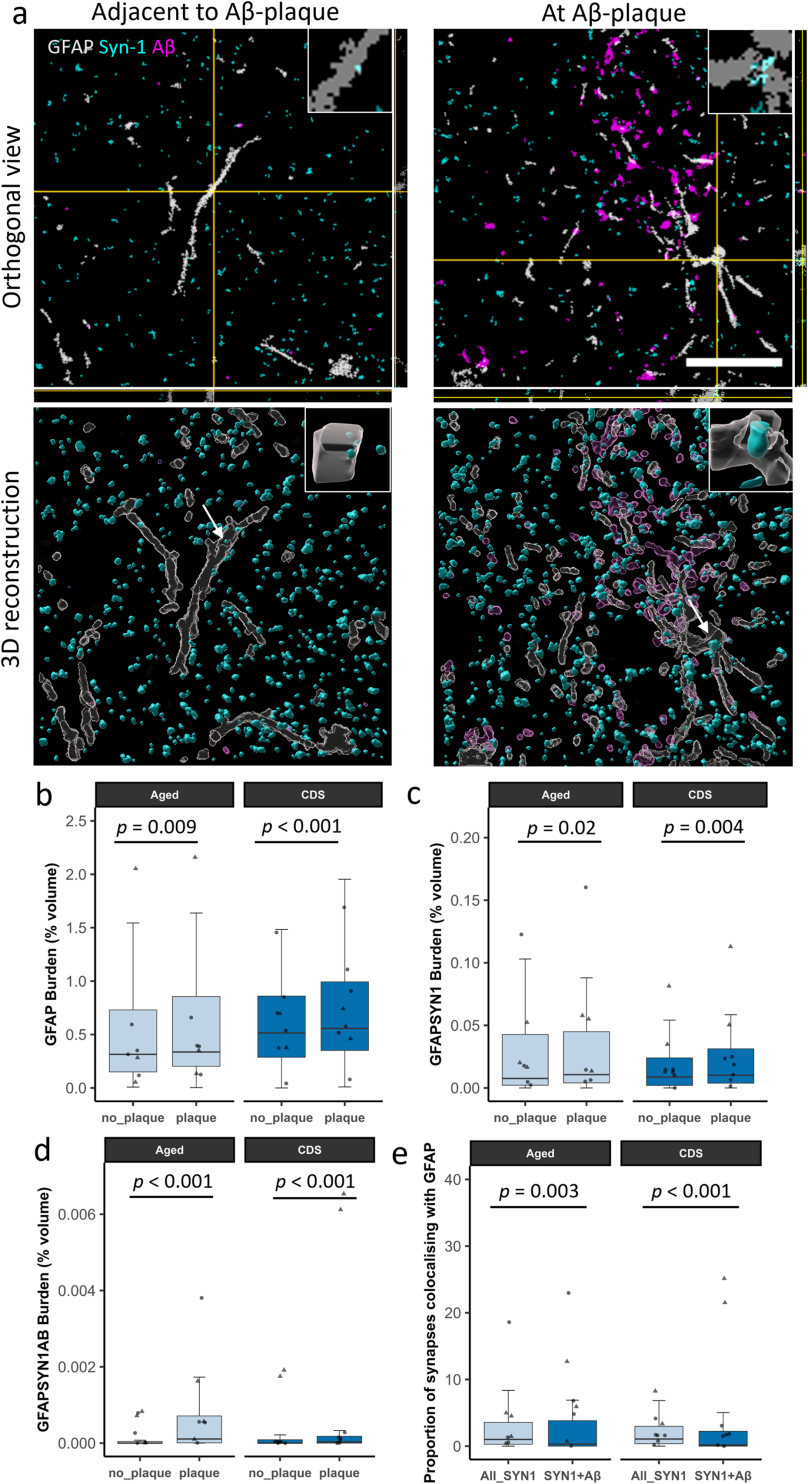
Amyloid‐beta pathology induces increased synaptic engulfment by astrocytes in the feline brain. (a) Segmented confocal images of cognitive dysfunction syndrome (CDS)‐affected feline parietal cortex, showing a region with amyloid‐beta pathology and a paired image acquired approximately one field of view away in the same cortical layer in an area without pathology. Tissue was immunostained for synapses (synapsin‐1, cyan), amyloid‐beta (Aβ, magenta) and astrocytes (GFAP, grey). Scale bar = 20 μm. Inserts = 5 μm × 5 μm. The top row of images shows orthogonal views. The bottom row of images shows 3D reconstructions generated using IMARIS and have been rotated along the *z*‐axis, to further demonstrate that synaptic protein is within astrocytes. Inserts show close up of colocalisation highlighted by arrow. (b–e) Colocalisation analysis reveals that the image stacks acquired in regions with amyloid‐beta pathology show increased astrogliosis (GFAP burden) (b), colocalisation between astrocytes and synapses (GFAP and synapsin‐1 burden) (c), and triple colocalisation between astrocytes, synapses and amyloid‐beta (GFAP, synapsin‐1 and amyloid‐beta burden) (d). Finally, when analysing all image stacks, synapses colocalising with amyloid‐beta are less likely to be engulfed by astrocytes (e). Boxplots show quartiles and medians calculated from each image stack. Data points represent case means (females = circles, males = triangles). Stats = LMEM (Variable ~ Plaque_vs_no‐plaque*Group + 1|Case/ROI_number). *p* Values were calculated from post hoc testing with Tukey correction for multiple comparisons.

Synaptic accumulation of amyloid‐beta and the engulfment of synapses by glial cells were assessed by immunostaining for amyloid‐beta (4G8), synapses (synapsin‐1), astrocytes (GFAP) and microglia (IBA1) (Figures 1a, 2a and 3a), along with the quantification of the percentage volume of the 3D image stacks occupied by each individual channel and the colocalisation between channels. First, we set out to investigate if amyloid‐beta accumulates within synapses in the aged and CDS cat brain. Linear mixed effect modelling of Tukey transformed data (Variable ~ Group + 1|Case) revealed that the amount of amyloid‐beta (4G8) staining was greater in the CDS (*t*(22) = 4.238, *p* < 0.001) and aged group (*t*(22) = 2.847, *p* = 0.024) when compared to the young group (Figure 1b). There was no difference between the CDS and aged group (*t*(22) = 1.667, *p* = 0.240). Similarly, there was increased colocalisation between synapses (synapsin‐1) and amyloid‐beta (4G8) in both the CDS (*t*(22) = 5.004, *p* < 0.001) and aged groups (*t*(22) = 3.701, *p* = 0.003) compared to the young controls, but no difference between CDS and aged group (*t*(22) = 1.616, *p* = 0.260) (Figure 1c).

### 3.2 Amyloid‐Beta Pathology Induces Increased Synaptic Engulfment by Microglia in the Feline Brain

To investigate if amyloid‐beta pathology may contribute to synaptic loss via the induction of aberrant synaptic pruning by microglia in the feline brain, a region of interest (ROI) was selected where there was amyloid‐beta pathology and then a paired ROI was selected approximately one field of view over in an area without amyloid‐beta pathology but still within the same cortical layer (Figure 2a). LMEM of the data (Variable ~ Plaque_vs_no‐plaque*Group + 1|Case/ROI_number) found that in regions of amyloid‐beta pathology there was microgliosis (increased IBA1 burden) in both the aged (*t*(122) = 2.798, *p* = 0.006) and CDS group (*t*(122) = 5.089, *p* < 0.001) (Figure 2b). In the aged (*t*(122) = 4.014, *p* < 0.001) and CDS group (*t*(122) = 4.780, *p* < 0.001) there was also increased internalisation of synapses by microglia (synapsin‐1 and IBA1 colocalisation) around amyloid‐beta plaques (Figure 2c). Further, near amyloid‐beta plaques we found increased internalisation of synapses colocalising with amyloid‐beta by microglia (synapsin‐1, 4G8, and IBA1 triple colocalisation) in both the aged (*t*(122) = 8.353, *p* < 0.001) and CDS group (*t*(122) = 8.678, *p* < 0.001) (Figure 2d)—suggesting that microglia may be engulfing synapses that contain amyloid‐beta. When analysing all image stacks, including those taken at an amyloid plaque and away from an amyloid plaque, we found that synapses colocalising with amyloid‐beta were more likely to colocalise with microglia in both the aged (*t*(243) = 5.974, *p* < 0.001) and CDS groups (*t*(247) = 15.464, *p* < 0.001) (Figure 2e). Finally, we compared the IBA1 burden, IBA1 and synapsin‐1 colocalisation, IBA1 and 4G8 colocalisation, and IBA1, 4G8 and synapsin‐1 colocalisation between the young, aged and CDS groups. In this analysis, the 10 image stacks with the highest levels of amyloid‐beta pathology were selected for each case. For all readouts, we found an increase in the CDS group compared to the young group. We found no differences between the aged group and the young group or the aged group and the CDS group (Supplementary Figure S1).

### 3.3 Amyloid‐Beta Pathology Induces Increased Synaptic Engulfment by Astrocytes in the Feline Brain

We observed astrogliosis, by way of increased GFAP burden, in areas with amyloid‐beta plaques when compared to regions without plaques in both the aged (*t*(122) = 2.666, *p* < 0.009) and CDS groups (*t*(242) = 4.644, *p* < 0.001) (Figure 3b). In the aged (*t*(122) = 2.360, *p* = 0.02) and CDS groups (*t*(122) = 2.907, *p* = 0.004), there was also increased colocalisation of synapses (synapsin‐1) with astrocytes (GFAP) around amyloid‐beta plaques (Figure 3c). Additionally, there was increased triple colocalisation between astrocytes (GFAP), synapses (synapsin‐1) and amyloid‐beta (4G8) around amyloid‐beta pathology in both the aged (*t*(122) = 6.969, *p* < 0.001) and CDS groups (*t*(122) = 5.193, *p* < 0.001) (Figure 3d)—again suggesting that astrocytes might be eliminating amyloid‐beta containing synapses. However, in contrast to what we found with microglia, synapses colocalising with amyloid‐beta were less likely to colocalise with astrocytes in both the aged (*t*(242) = 2.993, *p* = 0.003) and CDS groups (*t*(247) = 4.079, *p* < 0.001) (Figure 3e). Finally, we compared the GFAP burden, GFAP and synapsin‐1 colocalisation, GFAP and 4G8 colocalisation, and GFAP, 4G8 and synapsin‐1 colocalisation from all of the image stacks between the young, aged and CDS groups (Supplementary Figure 2). In this analysis, the 10 image stacks with the highest levels of amyloid‐beta pathology were selected for each case. When comparing between the CDS and young group, the CDS group had an increase in the burden of GFAP‐positive astrocytes (*t*(22) = 2.610, *p* = 0.041) (supplementary figure 2a), GFAP and amyloid‐beta colocalisation (*t*(22) = 3.610, *p* = 0.004) (supplementary figure 2d), and a trend for an increase in triple colocalisation between GFAP‐positive astrocytes, amyloid‐beta and synapsin‐1 (*t*(22) = 2.459, *p* = 0.056) (Supplementary Figure 2c). No other comparisons showed differences between groups.

Although aged and CDS cats had similar overall amyloid‐beta burdens (Figure 1b), the amyloid burden in each cat correlated with synaptic ingestion by microglia and astrocytes only in CDS, not in aged cats (Supplementary Figure 3), indicating that amyloid plaques are associated with more local damage in CDS than in healthy ageing.

## 4 Discussion

In this study, we observe that amyloid‐beta colocalises with synapses in the aged and CDS‐affected feline brain and is associated with regional gliosis, as well as increased synaptic engulfment by both microglia and astrocytes.

Our finding that amyloid‐beta colocalises with synapses in feline CDS is in line with previous findings in AD brains and rodent models of amyloid pathology (Koffie et al. 2009, 2012; Jackson et al. 2019; Pickett et al. 2019; King et al. 2023) and supports the hypothesis that amyloid‐beta accumulation contributes to behavioural and cognitive changes observed in feline CDS. The exact mechanisms by which amyloid‐beta induces synaptotoxicity remain a matter of debate. Data from preclinical studies and human post‐mortem tissue have implicated the interaction of amyloid‐beta with several synaptic proteins in mediating its synaptotoxic effects, such as cellular prion protein (PrPC), transmembrane protein 97, metabotropic glutamate receptor 5 (mGluR5) and NMDA receptors (Laurén et al. 2009; Larson et al. 2012; Folch et al. 2018; Colom‐Cadena et al. 2024).

Additionally, our finding that gliosis and increased synaptic engulfment by microglia and astrocytes occur in the vicinity of amyloid‐beta plaques further supports the hypothesis that amyloid‐beta exerts a pathological effect within the feline brain. From our data, we are unable to determine whether glia are removing degenerating or functional synapses. In support of the latter, data from model systems of AD suggest that increased synaptic engulfment by microglia leads to synapse loss and cognitive dysfunction. Importantly, blocking synaptic engulfment in these models rescues cognitive function (Hong et al. 2016; Shi et al. 2017; Bie et al. 2019). This indicates that microglia are engulfing functional synapses and thereby contributing to clinical progression. Furthermore, increased synaptic engulfment by microglia does not appear to be a common feature of neurodegenerative diseases, but rather a direct consequence of amyloid‐beta accumulation. We recently demonstrated increased synaptic engulfment by both astrocytes and microglia in AD (Tzioras, Daniels, et al. 2023). However, in the brains of individuals who died with the primary tauopathies progressive supranuclear palsy (PSP) (McGeachan et al. 2025) and frontotemporal dementia caused by the MAPT 10+16 mutation (Dando et al. 2024), we did not observe increased microglial engulfment, and increased astrocytic engulfment was only observed in PSP. Our data showing that synaptic engulfment is greatest in the vicinity of amyloid‐beta plaques, and the finding that amyloid‐beta‐containing synapses are more likely to be engulfed when compared to all synapses in the feline brain, further support this hypothesis. Therefore, feline CDS serves as a naturally occurring model of human AD, providing a valuable avenue for studying disease mechanisms and testing therapeutic interventions targeting amyloid‐beta‐induced synaptic loss. Studying feline CDS has the potential to enhance our understanding and management of feline CDS while simultaneously contributing to the development of treatments for human dementia.

For several pathological readouts, including microgliosis, astrogliosis and synaptic engulfment by microglia, our findings show an increase when comparing the CDS group to the young group. However, no significant differences were observed between the CDS group and age‐matched controls, nor between the aged and young groups. These results suggest that the pathological changes observed in CDS may arise from an interaction between ageing processes and these mechanisms, rather than being solely attributable to either factor independently. While the data highlight a potential link between these pathologies and CDS, the lack of distinction between CDS and age‐matched controls makes it difficult to conclusively attribute these changes as causal in the development of CDS. However, despite similar overall burdens between aged and CDS cats, we observed that synaptic ingestion by microglia and astrocytes correlated with amyloid‐burden only in the CDS group. This suggests that amyloid‐beta plaques exert greater toxic effects in CDS than in healthy ageing. This finding aligns with human studies, where amyloid presence alone does not fully predict disease severity, but its interaction with glial cells appears critical in driving pathology (De Strooper and Karran 2016). Additionally, one limitation of our study is the relatively small sample size. We used all available samples, but obtaining body donations from owners is a sensitive topic and presents a logistical challenge. It is possible that our study is underpowered to detect smaller effect sizes between the young and aged groups or the aged and CDS groups. Another possibility is the potential for undetected cognitive changes in the age‐matched control group. Although these cats had no reported history of neurological disease, subtle behavioural signs may have gone unrecognised or unreported. This highlights the need for more standardised behavioural and cognitive assessments in aged‐matched controls in future studies. Despite the lack of significance between CDS and aged cats in the measurements performed in this study, there is a stepwise increase in pathological features we measured between young, aged, and CDS animals and significant main effects of ANOVAs in many measures, indicating that further research with a larger sample size is warranted to explore this question in more depth.

The absence of precise PMI data represents another limitation, and we highlight the need for systematic PMI recording in future veterinary neuropathological studies. The resolution of confocal microscopy is limited by the diffraction limit of light, making it difficult to confidently resolve individual synapses. Future studies should aim to confirm the presence of amyloid‐beta within synapses and determine the proportion affected using higher‐resolution techniques, such as immunogold or correlative light and electron microscopy. We did not have access to appropriately prepared samples for such analysis.

In conclusion, our data provide insight into mechanisms by which amyloid‐beta pathology may lead to synaptic dysfunction and loss, revealing a potential link between age‐related amyloid‐beta deposition and the behavioural and cognitive changes observed in feline cognitive dysfunction syndrome.

## Author Contributions

**Robert I. McGeachan:** formal analysis, investigation, supervision, writing – original draft, writing – review and editing. **Lucy Ewbank:** formal analysis, investigation. **Meg Watt:** resources, writing – review and editing. **Lorena Sordo:** investigation, resources, writing – review and editing. **Alexandra Malbon:** resources, writing – review and editing. **Muhammad Khalid F. Salamat:** resources. **Makis Tzioras:** methodology. **Joao Miguel De Frias:** resources. **Fiona Houston:** resources. **Danièlle Gunn‐Moore:** funding acquisition, resources, writing – review and editing. **Tara L. Spires‐Jones:** conceptualization, formal analysis, funding acquisition, methodology, supervision, writing – review and editing.

## Conflicts of Interest

None of the authors have a direct conflict of interest to declare. In the interest of transparency, TSJ is a scientific advisory board member of Scottish Brain Sciences, Cognition Therapeutics, and Race Against Dementia and has consulted for Jay Therapeutics, AbbVie, Eisai and Sanofi, and MT is an employee of Scottish Brain Sciences.

## Peer Review

The peer review history for this article is available at https://www.webofscience.com/api/gateway/wos/peer‐review/10.1111/ejn.70180.

## Supporting information


**Figure S1.** Effect of ageing and CDS on microglia–synapse interactions in feline parietal cortex.
**Figure S2.** Effect of ageing and CDS on astrocyte–synapse interactions in the feline parietal cortex.
**Figure S3.** Correlations between amyloid beta burdens and synaptic ingestion by glia reveal more toxicity of plaques in CDS than aged controls.

## Data Availability

Upon acceptance for publication, all spreadsheets and statistical analysis files will be shared on Edinburgh Datashare. Raw images and data are available from the lead authors upon reasonable request.
