# Supplementary material for: Amyloid‐Beta Pathology Increases Synaptic Engulfment by Glia in Feline Cognitive Dysfunction Syndrome: A Naturally Occurring Model of Alzheimer's Disease
Source: Eur J Neurosci. 2025 Aug 11;62(3):e70180. doi: 10.1111/ejn.70180 (PMC12340200; doi:10.1111/ejn.70180)
Supplement: Supplementary file 1 — Figure S1. Effect of ageing and CDS on microglia–synapse interactions in feline parietal cortex. Figure S2. Effect of ageing and CDS on astrocyte–synapse interactions in the feline parietal cortex. Figure S3. Correlations between amyloid beta burdens and synaptic ingestion by glia reveal more toxicity of plaques in CDS than aged controls. [file EJN-62-0-s001.docx]

**Supplementary data**

**
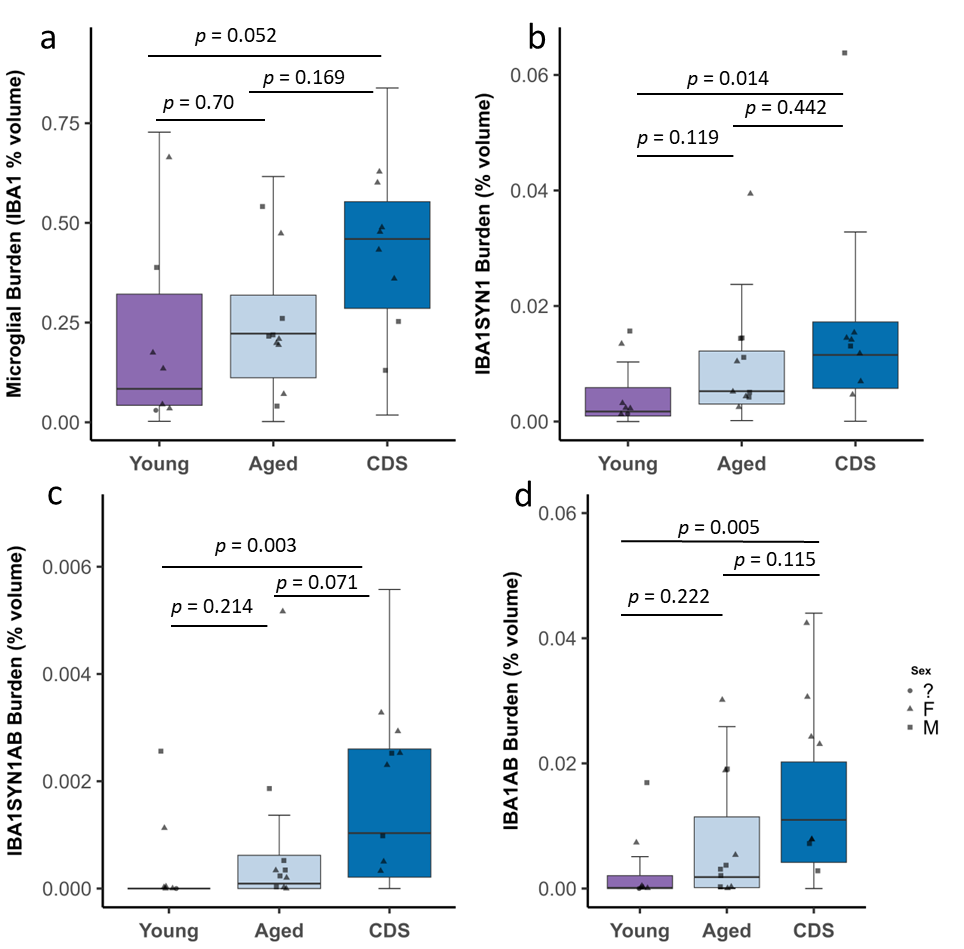
**

**Supplementary figure 1: Effect of ageing and CDS on microglia – synapse interactions in feline parietal cortex**

**a)** Quantitative analysis reveals a trend for an increase in IBA1 burden in CDS group compared to the young (*t*(22) =2.494, *p* = 0.052) but no difference when compared to the aged group (*t*(22) = 1.877, *p* = 0.169). There was no difference between aged and young group (*t*(22) =0.812, *p* = 0.70)
**b)** Quantitative analysis reveals an increase in IBA1 and synapsin-1 colocalisation in CDS group compared to the young (*t*(22) =3.113, *p* = 0.014) but no difference when compared to the aged group (*t*(22) = 1.243, *p* = 0.442). There was no difference between aged and young group (*t*(22) =2.074, *p* = 0.119)
**c)** Quantitative analysis reveals an increase in IBA1, synapsin-1 and amyloid-beta triple colocalisation in CDS group compared to the young (*t*(22) =3.796, *p* = 0.003) and a trend towards an increase when compared to the aged group (*t*(22) = 2.337, *p* = 0.071). There was no difference between the aged and young groups (*t*(22) =1.737, *p* = 0.214)
**d)** Quantitative analysis reveals an increase in IBA1 and amyloid-beta colocalisation in CDS group compared to the young (*t*(22) =3.548, *p* = 0.005 but no difference when compared to the aged group (*t*(22) = 2.089, *p* = 0.115). There was no difference between aged and young group (*t*(22) =1.716, *p* = 0.222)
**a-d)** Boxplots show quartiles and medians calculated from each image stack. Data points represent case means (sex unknown = circles, females = triangles, males = squares). Statistics: LMEM (variable ~ Group + 1 | case). *p*-values were calculated from post-hoc testing with Tukey correction for multiple comparisons.


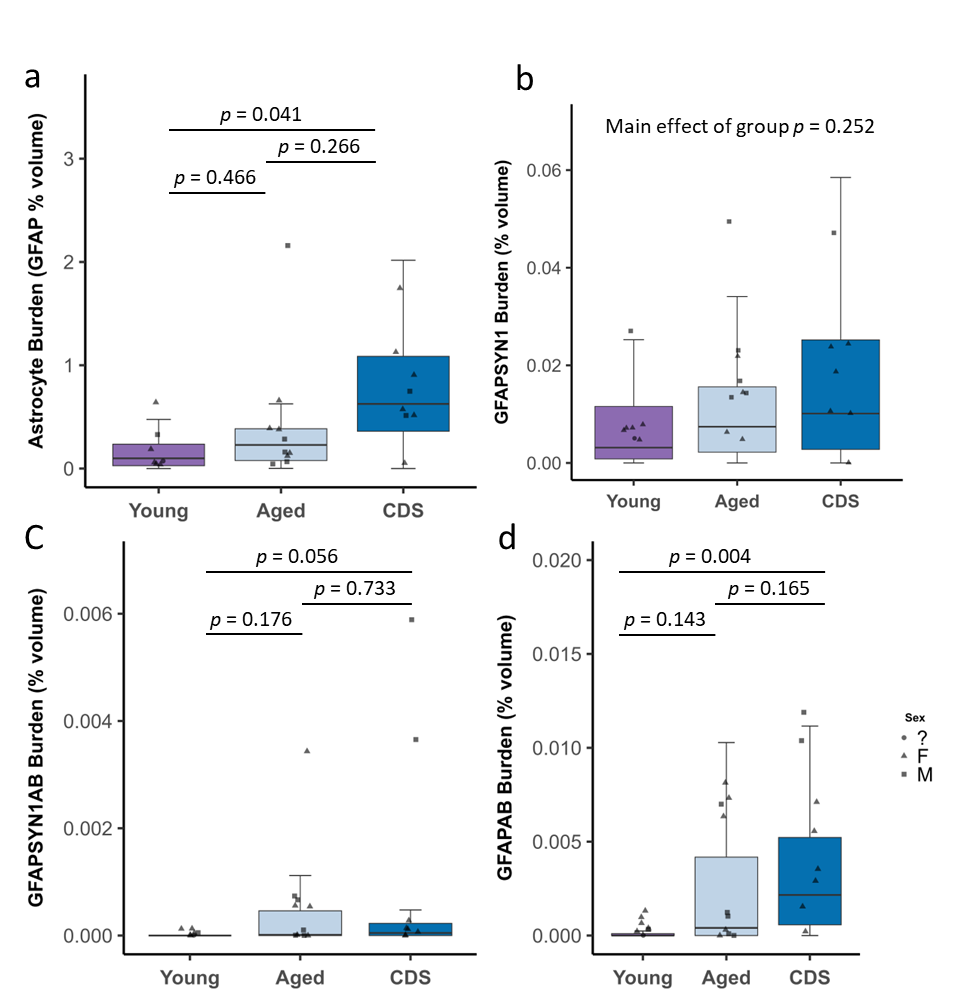


**Supplementary figure 2: Effect of ageing and CDS on astrocyte – synapse interactions in the feline parietal cortex**

**a)** Quantitative analysis reveals an increase in GFAP burden in CDS group compared to the young (*t*(22) =2.610, *p* = 0.041) but no difference when compared to the aged group (*t*(22) = 1.602, *p* = 0.266). There was no difference between aged and young group (*t*(22) =1.199, *p* = 0.466)

**b)** Anova after linear mixed effect modelling of the data reveals no main effect of group on the colocalisation between astrocytes and synapsin-1 (F[2/21.96] = 1.4674, *p* = 0.252). As there was no main effect, pairwise comparisons were not performed.

**c)** Quantitative analysis reveals a trend for an increase in GFAP, synapsin-1 and amyloid-beta triple colocalisation in CDS group compared to the young (*t*(22) =2.459, *p* = 0.056) but no difference when compared to the aged group (*t*(22) = 0.756, *p* = 0.733). There was no difference between the aged and young groups (*t*(22) =1.855, *p* = 0.176)

**d)** Quantitative analysis reveals an increase in GFAP and amyloid-beta colocalisation in CDS group compared to the young (*t*(22) =3.610, *p* = 0.004) but no difference when compared to the aged group (*t*(22) = 1.891, *p* = 0.165). There was no difference between aged and young group (*t*(22) =1.972, *p* = 0.143)

a-d) Boxplots show quartiles and medians calculated from each image stack. Data points represent case means (sex unknown = circles, females = triangles, males = squares). Statistics: LMEM (variable ~ Group + 1 | case). *p*-values were calculated from post-hoc testing with Tukey correction for multiple comparisons.


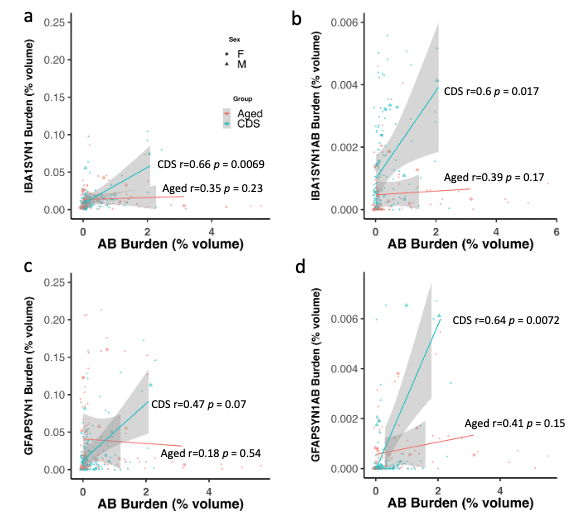


**Supplementary figure 3: Correlations between amyloid beta burdens and synaptic ingestion by glia reveal more toxicity of plaques in CDS than aged controls.**

Correlations between amyloid beta burden and burdens of (a) IBA1 stained microglia colocalised with synapsin-1 labelled pre-synapses (b) IBA1, synapsin-1 and amyloid-beta (c) GFAP and synaptophysin, and (d) GFAP, synapsin-1 and amyloid-beta show a positive correlations in CDS but not aged cats. Spearman correlation test results shown on each panel.
